# Cost-effectiveness of a health-social partnership transitional program for post-discharge medical patients

**DOI:** 10.1186/1472-6963-12-479

**Published:** 2012-12-24

**Authors:** Frances Kam Yuet Wong, June Chau, Ching So, Stanley Ku Fu Tam, Sarah McGhee

**Affiliations:** 1 School of Nursing, The Hong Kong Polytechnic University, Hunghom, Kowloon, Hong Kong, China SAR; 2 School of Public Health, Department of Community Medicine, University of Hong Kong, Hong Kong, China SAR; 3 Geriatric Team, Queen Elizabeth Hospital / Hong Kong Buddhist Hospital, Hong Kong, China SAR

**Keywords:** Health-social transitional care, Readmission, Cost-effective analysis

## Abstract

**Background:**

Readmissions are costly and have implications for quality of care. Studies have been reported to support effects of transitional care programs in reducing hospital readmissions and enhancing clinical outcomes. However, there is a paucity of studies executing full economic evaluation to assess the cost-effectiveness of these transitional care programs. This study is therefore launched to fill this knowledge gap.

**Methods:**

Cost-effectiveness analysis was conducted alongside a randomized controlled trial that examined the effects of a Health-Social Transitional Care Management Program (HSTCMP) for medical patients discharged from an acute regional hospital in Hong Kong. The cost and health outcomes were compared between the patients receiving the HSTCMP and usual care. The total costs comprised the pre-program, program, and healthcare utilization costs. Quality of life was measured with SF-36 and transformed to utility values between 0 and 1.

**Results:**

The readmission rates within 28 (control 10.2%, study 4.0%) and 84 days (control 19.4%, study 8.1%) were significantly higher in the control group. Utility values showed no difference between the control and study groups at baseline (p = 0.308). Utility values for the study group were significantly higher than in the control group at 28 (p < 0.001) and 84 days (p = 0.002). The study group also had a significantly higher QALYs gain (p < 0.001) over time at 28 and 84 days when compared with the control group. The intervention had an 89% chance of being cost-effective at the threshold of £20000/QALY.

**Conclusions:**

Previous studies on transitional care focused mainly on clinical outcomes and not too many included cost as an outcome measure. Studies examining the cost-effectiveness of the post-discharge support services are scanty. This study is the first to examine the cost-effectiveness of a transitional care program that used nurse-led services participated by volunteers. Results have shown that a health-social partnership transitional care program is cost-effective in reducing healthcare costs and attaining QALY gains. Economic evaluation helps to inform funders and guide decisions for the effective use of competing healthcare resources.

## Background

Patients who are discharged from the hospital and readmitted within a short time are a considerable cause for concern among healthcare providers. Rehospitalizations place a financial burden on hospitals particularly those using public funding. Multiple hospital admissions also tend to compromise patients’ quality of life. Many transitional care programs have been implemented to support patients after hospital discharge, and the effectiveness of these programs has been examined. Studies that produced positive results in controlling hospital readmissions and enhancing quality of life tend to share some common features. These more successful programs are in general comprehensive and well supported with a multi-disciplinary care team [1-3]. The nurse usually plays a pivotal role in the team, including in the provision of direct home-based or telephone follow-up care [4,5] and indirect care such as coordination. Care coordination involves negotiating care with multiple providers, making referrals and ensuring that the program is delivered in compliance with the set protocol [4,6]. Patient education is an essential element of these transitional care programs that are targeted at reinforcing health behavior, empowering self-care, enhancing treatment adherence, and monitoring signs and symptoms [5,7]. The intervention provided is proactive, continuous and regular [2,8]. Wong et al. [6] summarized the key features of the transitional care described above with 4Cs, which are comprehensiveness, continuity, coordination and collaboration.

The current literature has provided substantial evidence to support the thesis that transitional care programs can produce positive outcomes in regard to the reduction of readmission rates and the enhancement of quality of life and care satisfaction [4,6,9]. However, cost is not usually included as one of the outcome measures. When cost is included, the study often simply reports the expenditure and possible cost savings in a descriptive manner. Ledgwidge et al. [10] provided multidisciplinary care for heart failure patients for 3 months, and the cost of the service hospitalization prevented was estimated to be €586. Another study using geriatricians to target medication review, self-disease management and communication among health professionals achieved a cost saving of €519 per participant for a 6-month follow-up [11]. Naylor et al. [4] used advanced practice nurses to provide home follow-up for elderly patients for 3 months, finding significantly lower healthcare costs for the intervention group and yielding estimated mean cost savings of US$4,845 per patient. Kwok et al. [12] also used nurse home visits provided to elderly patients for 6 months and reported a marginal difference (p = 0.048) of healthcare costs between the intervention and control group, with a median difference of US$2024. Miller et al. [13] introduced early discharge supported by home visits and rehabilitation service for older people, and the cost for the intervention group was £1727 (p = 0.054) lower than that for the usual group. Wong et al. [2] similarly used nurse specialist follow-up for early discharged diabetic patients and was able to reduce the length of stay by 3.7 days (p < .001) and saved US$1633 per patient.

There is a paucity of studies executing full economic evaluation, which considers both cost and outcomes [14], for transitional care programs. Amongst the few studies that have been published, Patel et al. [15] conducted an analysis using the outcome measures of cost and quality-adjusted life year (QALY) gain when comparing the effects of domiciliary care for stroke patients with stroke unit or stroke team care. Similar QALY gains were found among the three groups, but there was a 59% probability that domiciliary care was cost-effective. The mean healthcare and social care costs for stroke unit, stroke team and domiciliary care were respectively £11450, £9527, and £6840 for over 12 months. Stewart et al. [16] performed an economic modeling for estimating the cost savings achieved by three different types of UK-wide programs for patient follow-up using specialist nurse management. The analysis was based on existing and projected healthcare data and cost. The calculated expenses for home-, clinic- and home- plus clinic-based follow-up were £69.4, £73.1 and £72.5 million per annum respectively. The programs were estimated to be able to bring about 38.5% (home-based), 40.6% (clinic-based) and 40.3% (home plus clinic-based) reduction in recurrent bed utilization. It was projected that a home-based program using specialist nurse management could bring about annual savings equivalent to £169000 per 1000 patients treated.

Based on the available literature, it is uncertain whether transitional care programs especially those adopting a health-social partnership approach, are cost-effective in supporting patients returning to the community after hospitalization. Many of the studies focused on clinical outcomes only. This study is therefore launched to fill this knowledge gap by examining the cost-effectiveness of a health-social partnership transitional care program for patients discharged from hospitals.

## Methods

### Ethics

The study protocols were reviewed and ethical approval was obtained from the Research Ethics Committee of the study hospital (Reference KC/KE-08-0990/ER-5) and the Human Subjects Ethics Sub-committee at the university with which the principal investigator was affiliated (Reference HSEAR20050920001). All potential subjects have received full explanation of the study and the agreed participants had to sign a consent form. The confidentiality and anonymity of the participants were protected and all data were identified by case numbers only.

### Randomized controlled trial

This study was conducted alongside a randomized controlled trial. The details of the study have been described elsewhere [6]. Briefly, it involved medical patients discharged from an acute regional hospital in Hong Kong who met specific inclusion criteria, namely (a) being aged 60 or above, (b) MMSE >20, (c) ability to speak Cantonese, (d) living within the hospital service area, and (e) ability to be contacted by phone. The exclusion criteria were (a) having been discharged from institutional care, (b) being followed up by designated disease management programs, (c) inability to communicate, and (d) dying. The subjects who consented to participate were randomized into control and intervention groups. The control group received usual discharge care and the intervention group received both usual care and a health-social partnership transitional care management program (HSTCMP) at discharge.

The HSTCMP was a 4-week program with weekly planned events delivered by the nurse case manager (NCM) and trained volunteers (TV), guided by protocols and structured documentations. Both the NCM and TVs have received training to equip them to provide the interventions. In week 1, the NCM and the TV would conduct a home visit together. The NCM would conduct an assessment in the domains of environment, physical, psychosocial and health-related behaviors and provide relevant intervention based on the Omaha System. The Omaha System is originally developed in the United States and used widely in community care [17]. The research team has used the Omaha System in the local community and found it comprehensive and valid for use in the local context [6]. In supporting the NCM, the TVs would provide social support to the patient mainly in the emotional dimensions by expressing concerns and showing support [18]. The TVs had a resource kit on healthy home environment and available community resources if they need to provide some relevant information to the patients. At the end of the visit, the NCM would set mutual goals with the patient to enhance health. In week 2, the NCM would make a follow-up telephone call based on the Omaha System and review the mutual health goals set in week 1. In week 3, the TVs would conduct a home visit in pairs. The social visit was to show emotional support to the patient and see if there were needs for referrals to the social workers for further social assessment and interventions such as daily living assistance, housing assistance, and counseling. In week 4, the NCM would make the final telephone call to the client. The NCM would assess the client’s health needs, monitor progress, provide health advice, reinforce health self-management behavior, assess the need for referral, and review the health goals with the patients. The NCM assumes the overall responsibility in the transitional program, and is supported by the TVs and social worker in the social aspects. The health-social care team and the researchers held regular case reviews to ensure that the intervention was delivered according to the set protocols and to discuss issues of concerns.

### Cost and health outcomes

The cost and health outcomes were compared between the patients receiving the HSTCMP and usual care. The economic evaluation only involved direct costs from healthcare providers and patients. Indirect costs such as productivity loss were not considered to be relevant, since most patients were either retired (94%) or not in full-time employment (4%). Data on health services utilization, including the number of readmissions and length of stay of each admission, were extracted from the hospital information systems. Quality of life (QOL) was measured with the Hong Kong Chinese version of the 36-item Short-Form Health Survey (SF-36 HK). Using a locally developed algorithm [19], we transformed the health states described by the SF-36 HK into utility values between 0 and 1. These utility values could be used to calculate the quality-adjusted life years (QALYs) when multiplied by the length of time spent in that health state. For patients who missed either one of the follow-up visits, the missing QOL values were imputed using a regression equation derived from all patients with complete QOL data, adjusted for age, gender and treatment group. Other missing follow-up data were replaced with the group means. The average QALYs gained in 28- and 84-day periods were estimated based on the change from baseline utility over the relevant period. Net monetary benefit, number of QALYs gained from the intervention and costs per QALY gained were calculated. The costing was based on the most current prices available and took a societal perspective. Total costs in the study group included the pre-program cost, program cost, and healthcare cost due to re-admission and associated accident and emergency room attendance, while for the control group, only healthcare cost was included.

#### Pre-program cost

The pre-program cost included the training time spent by the volunteers and staff in preparation for the delivery of the intervention. The unit cost of training for volunteers and staff was estimated by multiplying the duration of training with the hourly pay based on the corresponding salary. The total costs were estimated by multiplying the unit cost for the training with the number of volunteers trained or number of staff needed for the training. The monetary values for volunteers were estimated referencing the median salary and weekly hours of work from the Census and Statistics Department [20]. As for the staff, the appropriate hourly salary levels from the Master Pay Scale [21] were used for computation.

#### Program cost

The program costs included the estimation of patient and staff time spent during intervention following the standard protocol. The unit costs of the program for patient and staff were estimated by multiplying the total time of intervention for each case with the hourly pay based on the corresponding salary. The total costs were estimated by multiplying the unit cost by the total number of cases in the intervention program.

#### Healthcare cost

The healthcare cost included all direct medical costs due to readmission, which were calculated by multiplying the unit cost of HK$3650 (US$1 = HK$7.75) per hospital day with the length of stay for the readmission. Only readmissions related to index admission were counted. An addition of HK$820 [22] was added if the patient was admitted through the Accident and Emergency department. The total healthcare cost was therefore taken as the sum of the total bed day costs and the total cost of emergency admissions.

### Cost-effectiveness analysis

The bootstrap method was used to estimate confidence intervals for the difference in health outcome and cost between the two groups. It is an appropriate method of choice when the sampling distribution is unknown [23], and this is true in our case where the difference in health outcome and cost between groups is not certain. To estimate the cost-effectiveness of the intervention, the cost and QALYs gained at 28 and 84 days were estimated for both groups. Incremental cost-effectiveness ratios (ICER) between the groups were calculated by dividing the difference in cost by the difference in QALYs.

### Sensitivity analyses

Uncertainties around the parameters were tested with one-way and probabilistic sensitivity analyses. In the one-way sensitivity analysis, intervention cost and readmission rate were tested with a 30% variation to the base value for each item. For the readmission cost, we varied the length of stay (LOS) of re-admission episodes with a minimum and maximum value of the 95% confidence intervals of the LOS for the study and control groups separately. The overall LOS of re-admission episodes from all patients was also tested to assess the impact when the difference between the groups was ignored. In the probabilistic sensitivity analysis, random values for all parameters were selected from appropriate distributions for each parameter. The pre-program and program cost and readmission rate were varied within the range of ±30% of the base values using a uniform distribution. The LOS and utility scores were varied by fitting uniform distributions within the 95% CI of the values obtained in the study. ICERs were generated 1000 times with a random value for each parameter every time. The results were plotted on a cost-effectiveness plane and displayed with cost-effectiveness acceptability curves. All analyses were carried out using Microsoft Excel 2007 and STATA 10.1.

## Results

Among the 555 patients recruited, 283 received usual discharge care and 272 received both usual care and the health-social partnership transitional care management program. The readmission rates within 28 (control 10.2%, study 4.0%) and 84 days (control 19.4%, study 8.1%) were significantly higher in the control group. There was no significant difference in the LOS at readmission per patient between groups at 28 and 84 days. Utility values showed no difference between the control and study groups at baseline (p = 0.308). There was a significant within-group difference for both the study group (p < 0.001) and the control group (p = 0.011) over time. However, when compared between groups, utility was significantly higher for the study group than the control group at 28 (p < 0.001) and 84 days (p = 0.002). The study group also had a significantly higher QALYs gain (p < 0.001) over time at 28 and 84 days when compared with the control group (Table 1).

**Table 1:** Patient health outcomes

		**Study group (N = 272)**	**Control group (N = 283)**	**P-value**
Patient readmission rate (N, %)	28 days	11, 4.0%	29, 10.2%	0.005^a^
	84 days	22, 8.1%	55, 19.4%	<0.001^a^
Readmission LOS	28 days	2.7 (1.4, 4.1)	5.0 (2.6, 7.3)	0.456^b^
(Mean, 95% CI)	84 days	4.5 (2.6, 6.4)	6.0 (4.0, 8.0)	0.607^b^
Utility	Baseline	0.723 (0.707, 0.739)	0.735 (0.719, 0.752)	0.308^c^
(Mean, 95% CI)	28 days	0.764 (0.748, 0.781)	0.727 (0.710, 0.744)	<0.001^c^
	84 days	0.778 (0.762, 0.795)	0.751 (0.734, 0.768)	0.002^c^
	Within group comparison	<0.001^d^	0.011 ^d^	
QALY gain from baseline	28 days	0.0016 (0.0010, 0.0022)	−0.0003 (−0.0009, 0.0003)	<0.001^b^
(Mean, 95% CI)	84 days	0.0089 (0.0060, 0.0117)	0.0003 (−0.0024, 0.0030)	<0.001^b^

^a^ Pearson's chi-squared test,

^b^ Mann–Whitney U-test,

^c^ ANCOVA test (28 days and 84 days adjusted by baseline),

^d^ Repeated measures ANOVA.

The intervention cost, which included the pre-program training, staff and patient costs, was HK$1225 per subject for the study group (Table 2). The cost of readmission per subject within 28 and 84 days was lower in the study group than in the control group, and the differences were -HK$1505 (95% CI: -$2670, -$555) and -HK$3000 (−$5104, -$1211) for the two time periods respectively.

**Table 2:** Cost of intervention and healthcare services

		**Study case**			**Control case**	
**Intervention costs**	**N**	**Unit cost** ($)	**Total cost** ($)			
Pre-program training cost						
Volunteers	251	456	114408			
Social worker (SWA)	2	194	388			
Nurse (RN)	3	633	1899			
Program cost						
Nurse case manager (NO)	272	467	127159			
Volunteer	272	131	35508			
Extra social services	22	251	5528			
Patient time cost	272	178	48360			
**Readmission costs**	**N**	**Unit cost** ($)	**Total cost** ($)	**N**	**Unit cost** ($)	**Total cost** ($)
Emergency admission						
28 days	13	820	10660	31	820	25420
84 days	28	820	22960	63	820	51660
Bed day cost						
28 days	30	3650	109500	144	3650	525600
84 days	100	3650	365000	329	3650	1200850
Total readmission cost per subject :						
28 days			442			1947
84 days			1426			4426

The intervention resulted in cost savings at both 28 and 84 days, and there were gains in QALYs of 0.002 and 0.009 respectively (Table 3). One-way sensitivity analyses showed that raising the intervention cost or reducing the readmission rate and length of stay for both groups by 30% would increase the ICERs at 28 days, by up to HK$200,000 per QALY, whereas the ICERs at 84 days remained cost saving in all one-way sensitivity analyses.

**Table 3:** Main results and one-way sensitivity analyses of cost-effectiveness analysis

	**28 days**			**84 days**		
	**Incremental cost** ($)	**QALY gained**	**ICER** ($)	**Incremental cost** ($)	**QALY gained**	**ICER** ($)
Base	−280	0.002	−148828	−1774	0.009	−205079
Intervention cost						
30% lower	−648	0.002	−344111	−2142	0.009	−247561
30% higher	87	0.002	46455	−1407	0.009	−162596
Length of stay						
95% CI minimum	389	0.002	206455	−910	0.009	−105160
95% CI maximum	−949	0.002	−504111	−2639	0.009	−304997
Overall	190	0.002	100753	−1180	0.009	−136423
Readmission rate						
30% lower	171	0.002	91104	−874	0.009	−101072
30% higher	−732	0.002	−388760	−2674	0.009	−309085

**Figure 1:**
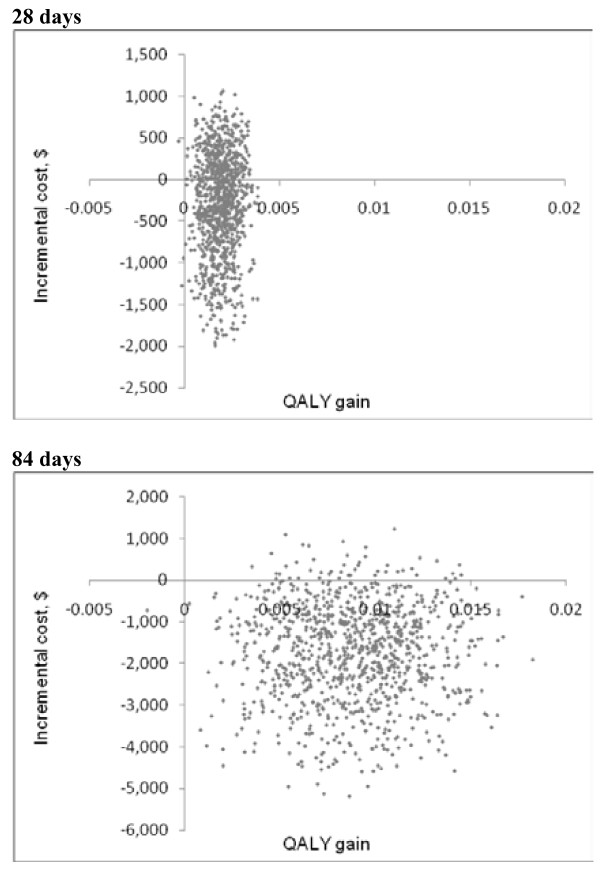
Cost-effectiveness plane.

**Figure 2:**
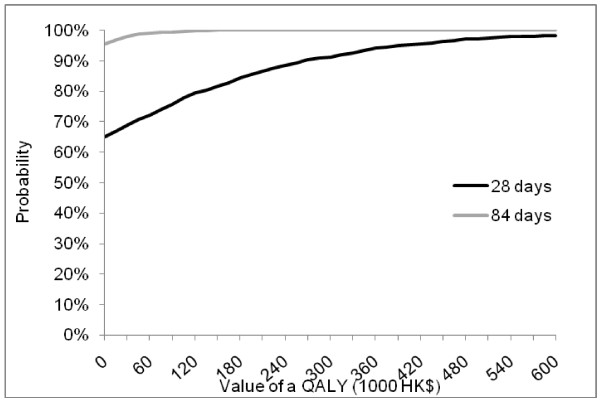
Cost-effectiveness acceptability curves.

The cost-effectiveness plane in Figure 1 shows that the intervention has a 65% and 95% chance of being cost saving at 28 and 84 days respectively. Cost-effectiveness acceptability curves in Figure 2 show that the intervention has an 89% chance of being cost-effective at the NICE threshold of £20000 (HK$240000, £1 = HK$12) [24].

## Discussion

This paper is original and contributes to the literature by providing evidence to show that a health-social partnership transitional care program is cost-effective in reducing healthcare costs and attaining QALY gains. Previous studies have provided evidence to support that transitional care can help reduce hospital readmissions and enhance clinical outcomes. However, these interventional programs tend to use healthcare professionals as the sole providers. There is no study that could be identified using volunteers in transitional care programs. There were programs that used volunteers or lay persons to provide support for patients, either hospital-based or community-based, and found them helpful to provide support to the patients. Based on findings from previous evidence which showed that transitional care model is effective and that volunteers can be conducive to patient outcomes, we built a health-social partnership transitional care model. There is a paucity of studies executing a full cost-effective analysis on transitional care model. This study fills the knowledge gap and demonstrated that the health-social partnership transitional care program is cost effective and brings about QALY gains.

The contemporary trend in post-discharge support services advocates health and social partnership [25,26] because clients returning home require different types of social support. Faulkner & Davies [18] outlined four types of social support: instrumental support, involving the provision of tangible resources to alleviate difficulties; appraisal support, which helps individuals to evaluate the impact of situations; informational support, which provides individuals with information to deal with problems; and emotional support, which enhances self-esteem and encouragement. In this study, the nurse case managers provided all four types of support, and the volunteers backed up by the social workers provided informational and emotional support [6]. The literature has reported studies that involved volunteers in patient programs, but the findings are limited to the descriptive level. Karwalajtys et al. [27] have reported using volunteer peer educator in the community to enhance cardiovascular health awareness. Sandhaus et al. [28] have involved volunteers to help reduce delirium among elderly in the hospital. Both studies remarked that the use of volunteers is a low-cost method of providing sustainable support to patients and the volunteers were welcomed by patients, their families, and nursing staff. However, no studies can be identified that report either health outcomes or cost-effectiveness when volunteers are included in the patient support programs. The integration of health and social care services as a newly-developed initiative needs the support of evidence to convince policy-makers of its value in both health outcomes and cost [29]. With the demand for healthcare resources across competing programs, cost analysis in health care is essential [30].

Of all healthcare expenses, hospital use occupies the major part of the expenditure [16]. Readmission rate is a commonly used outcome in health services research, and some studies have included cost as one of their outcome variables. How do the cost-related outcomes reported in those studies compare to this study? Since the content and length of the intervention programs, as well as the type and number of providers, vary among studies, it is very difficult to make a fair comparison of the results. Also, the intervention cost varies in different healthcare contexts. The following descriptive review will, however, help provide a synopsis of the cost savings in different places, thus helping readers to appreciate the extent of the cost savings reported in this study.

In a 12-week program with regular telephone contacts and education for heart failure patients after hospital discharge in Ireland, the cost was €5860 per patient. The intervention produced a net cost saving of €37,216 for 51 patients over 3 months [10]. Another 12-week transitional post-discharge care program introduced in the United States using advanced practice nurses also to follow up heart failure patients saved US$4,845 per patient over a year, with the intervention and control group spending respectively US$7,636 and US$12,481 [4]. A 6-month program was introduced to a group of elderly patients after leaving the hospital by geriatricians targeting risk factors for preventable readmissions in France. The mean cost of the intervention was €278 per patient, and the cost savings balanced against the cost of the intervention was €519 per patient [11]. Kwok et al. [12] provided regular nurse home visits also for 6 months to discharged elderly patients in Hong Kong, and the intervention cost was US$309 per subject. The mean total public health expenditure was reported to be lower in the intervention group, with a saving of US$2024 per patient. For a longer program of 12 months adopted for a group of post-discharge stroke patients involving health and social care in England, the cost of domiciliary care was £6840, which was cheaper when compared to the other two modes of interventions implemented by the stroke unit (£11450) and stroke team (£9527). The mean QALY gained were respectively 0.297, 0.216 and 0.221 for stroke unit, stroke team and domiciliary care, and there was a 59% probability of domiciliary care being cost-effective [15]. Another 1-year telephone support program was provided to community diabetes patients registered in a district in England, with the intervention estimated to cost £43000/QALY and to have a 29% probability of being cost-effective when measured against a threshold of £30000/QALY [31] All of the above programs used healthcare professionals as key service providers. The study by Richardson et al. [32] in England is one of the few that evaluated a program involving non-health professionals, a layperson-led self-care group. The group acted as expert patients, teaching other patients self-care support skills for long-term conditions in six weekly sessions in the community. The intervention was found to be associated with better patient outcomes at a slightly lower cost. There was a 0.020 QALY gain of the intervention group when compared with the control group, and a probability of 94% of being cost-effective when the value of £20000/QALY was considered.

This study spent HK$1225 (=US$158) per patient for the study group, and the cost of readmission per subject was reduced by HK$1490 (=US$192) and HK$2970 (=US$383) respectively at 28 and 84 days. The intervention had an 89% chance of being cost-effective when checked against the NICE threshold of £20000/QALY [23]. The expenditure figures reported in this study were substantially lower than in any of the studies reported above, and there was a high probability that the HSTCMP was effective when using the NICE threshold.

## Conclusions

Studies conducted to examine the effects of a transitional care program for hospital discharged patients mainly used healthcare professionals as key providers, and the outcome measures were confined to clinical and health outcomes. This study is original in conducting a cost-effectiveness analysis that takes into account cost and outcomes of a health-social partnership transitional care model. The results supported that a program that integrates the efforts of health and social care can be cost-effective. The use of trained volunteers to support the social aspects of care helps to contain costs and produce positive outcomes. Health-social partnership is regarded as an important trend of development for post-discharge services [33]. In some countries, such as United Kingdom [34] and Sweden [25], there are national policies in place that drive and regulate health and social care integration. However, there is no reported study that helps inform the policy makers if the health-social partnership model is sound clinically and economically. This study fills the knowledge gap and adds to the literature by providing evidence to inform healthcare managers and funders that health-social transitional programs are cost-effective.

There were several limitations to this study. First, it did not include nonmedical costs such as transport costs, carer costs and the like, which may have resulted in underestimated costs from a societal perspective. Second, this study only included expenditure on hospitalization; other medical costs such as outpatient and emergency room visits, treatment, investigations, and drugs were not considered. Third, only QALY gains at 28 and 84 days after hospital discharge were examined, and there was no data available after 84 days. The sustained cost-effectiveness of this program cannot be established.

## Competing interests

The authors declare that they have no competing interests.

## Authors’ contributions

FKYW designed, implemented the study and drafted the manuscript, JC, CS and SM performed the statistical analysis, drafted the results part of the manuscript, SKFT participated in the design and implementation of the study and helped to draft the manuscript. All authors read and approved the final manuscript.

## Pre-publication history

The pre-publication history for this paper can be accessed here:

http://www.biomedcentral.com/1472-6963/12/479/prepub
